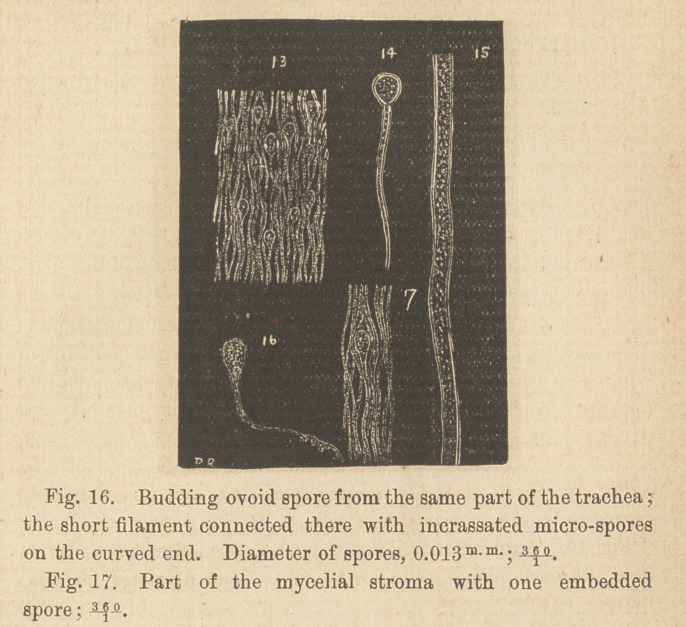# The Vegetable Nature of Croup

**Published:** 1883-05

**Authors:** Ephraim Cutter

**Affiliations:** New York City


### THE VEGETABLE NATURE OF CROUP.

A PAPER READ BEFORE THE AMERICAN SOCIETY OF MICROSCOPISTS, AT ITS ANNUAL MEETING, AT ELMIRA, AUGUST, 1882.

BY EPHRAIM CUTTER, M. D., NEW YORK CITY.

January 21, 1879, Clarence, aged four years and six months, child of Major E. F. and Abby F. Wyer, of Woburn, Mass., (near Boston), was taken with croup at supper-time. Always well before, save a lung fever in December, 1878, whooping cough in July, 1878, and an attack of simple croup on the 14th of January, 1879. The prominent symptoms were dyspnea of a dreadful character, aphonia constant; air exhaled readily, but inhaled with difficulty; body-surface not hot or feverish, and pulse not much quickened; respiration hurried. He was vomited and put on the ordinary means of relief, including the sub-sulphate of mercury. Though there was no membrane visible in the throat, still he grew rapidly worse, and on the 23d, while I went for tracheotomy tube, he died.

The parents were unusually willing to have all possible light thrown upon the case, so a *post-mortem* examination was readily granted, and performed on the next day in the presence of Drs. S. W. Kelley, G. W. Graves, A. P. Woodman, G. P. Bartlett and J. M. Harlow, all then of Woburn. I found the larynx filled with a thick, tenacious mass, that appeared to be distinct from anything else above or below it. On removing this plug it was found to be only about half an inch in length and thickness. Microscopically examined, the physicians assisting were unanimously of the opinion that it was made up of a true animal membrane, and was decidedly not vegetable in its nature and structure. On this ground it was reasoned that we were dealing with croup, and not diphtheria. The larynx and trachea were removed, and with a clinical microscope of the Boston optical works, which embraced a $\frac{1}{5}$ inch objective, three systems of lenses, 180° of angular aperture, and a one-inch eye-piece, making an amplification of about 400 diameters, I examined the plug and its membrane, with scrapings also from the trachea and secondary bronchi. *In all I found mycelial fungus filaments, single and in skeins of beauty.* From this examination I was led to change my opinion as to the animal character of the membrane. The gentlemen present, not being experts in the use of the microscope, of course had no opinion to express different from that already announced.

Hence, in view of the great importance, in a clinical point of view, of settling the real nature of the membrane, I employed the eminent algologist, Prof. Paulus F. Reinsch, of Erlangen, then temporarily sojourning in Boston, to study the pathological products found in the larynx and trachea, botanically and histologically. Immediately, he detected the mycelial filaments, and then went to work to cultivate the fungus, so that he might be able to identify it by the fructification. He also examined the tissues of the *post-mortem* specimen. It will be seen by his report that he agrees with me in my examination; *so that there are two witnesses to testify to the nature of this apparently animal membrane being a vegetable structure.* If this is the case in all instances of croup, the importance of this contribution is at once apparent.

REPORT BY PROF. P. F. REINSCH.

The larynx and trachea bear a remarkable fungoid vegetation, belonging to three, or at least two, different fungi. In the upper part of the larynx are prevalent cells of more rounded form, doubtless different states of evolution belonging to one or two different species of *Hyphomycetes.* The lower part of the larynx, as well as the trachea, was found overgrown with filamentaceous cells, inclosing short, rounded cells, resembling very much the mycelium, with interspersed spores characteristic of the *Mucorineæ.* The parasitic vegetation is found to be composed as follows:

*First*—Isolated spherical cells of .015 $^{m.m.}$ diameter, with constantly one nucleus; the plasm densely granulated.

*Second*—Two celled bodies, composed of smaller transverse elliptical cells, inclosed in the same involucrum; sometimes one cell is larger.

*Third*—Cellular bodies composed of four elliptical cells, forming short, rounded filaments.

*Fourth*—Spherical, four-celled bodies, composed of elliptical cells, mostly irregularly connected.

*Fifth*—Larger, irregular or tetrangular cells, with thick, laminated covering, with densely granulated plasm of reddish brown color.

*Sixth*—Short filaments, composed of short elliptical cells, on both sides, shortly angulated, with distinct nuclei; short two or more celled branchlets, of different shape and size, are mostly attached to them.

*Seventh*—Irregular, lancet-shaped cells, attenuated on both ends, constantly one distinct nucleus; plasm with larger transparent granules.

*Eighth*—Smaller elliptical cells, connected with hair-like filat ments; several cells sometimes are accumulated together on shory branchlets.

*Ninth*—Curved, lancet-shaped cells, three to five attached together, connected with filaments, the whole body resembling very much, budding two or more celled spores of *Hyphomycetes.*

*Tenth*—Undivided, long mycelial filaments, with distinct membrane and densely granulated contents, forming a tissue-like stroma, spread out all over the surface of the trachea, the lower part of the larynx, as well as within the mucous membrane, so that a transverse section of the lower part of the larynx, shows the peripherical discolored stratum of nearly 2 m.m. height, to be composed only of entostromatic filaments and elliptical spores, intermixed with fibular epithelial elements belonging to the mucous membrane. Spores of 0.013 m.m. diameter and ovoid circumference are frequently found interspersed between the fungoid filaments; spores and filaments, without any doubt, biologically connected.

REMARKS.

The most important point in this expert report is that the vegetable nature of the substance of the membrane is clearly made out. To be sure, some of the epithelial elements are distinguished in connection with the vegetable growths. Still it must be remembered that the host in this case is an animal, to-wit, man. So that it would be expected to find some animal tissues in the membrane. While it would not be safe to say that all croupal membranes are vegetable, still these examinations go to show that when examined by those who are acquainted with the subjects of micrographical botany, they may prove to be vegetable, as here.

The present was a rare opportunity to have such an authority study the morphology of the membrane in croup. As far as it goes, we can see no reason to doubt his decision and the satisfactory nature of the report. We conclude then:

*First*—That the inflammatory processes were secondary to the vegetation.

*Second*—The report shows that the vegetation penetrated the tissues of the larynx and trachea below.

*Third*—Regrets were expressed at the post-mortem examination that tracheotomy was not performed, but the fact that the vegetation was imbedded in the parts below the point of obstruction, shows that tracheotomy might not have been a success.

*Fourth*—If anything can be learned from this teaching, it shows the necessity of attacking the disease from the vegetable point of view. It would rather prohibit the pushing of anti-phlogistic measures, since the inflammation must be secondary, not primary.

*Fifth*—We should then perhaps rely on parasiticidal means, such as inhaling the fumes of burning sulphur, sprays of salicylic, carbolic and thymic acids, putting into the system as much food as possible, that is, good chemical, physiological and nerve food, easily assimilated and digested, so that the system can bear up in the fight. Or the sulphate of quinine might be sprinkled on the tongue —one grain once an hour or two hours, as shown with much success by Dr. Salisbury in diphtheria and scarlet fever.—*Vide essay.*

*Sixth*—The expert results were not indicated by any "leading strings." Indeed, it was rather a surprise to the medical gentlemen present that their decision as to the nature of the membrane should be so summarily upset, and yet they are a good average representation of the talent that has to handle such cases in the clinical point of view, on whom human lives depend, medically speaking.

*Seventh*—This case shows up how much we have to learn as to a disease that is so fatal and dreadful, and the need of more use of the microscope.

Finally, it is to be hoped that Prof. Reinsch may have other opportunities to study this disease further.

## Figures and Tables

**Figure f1:**
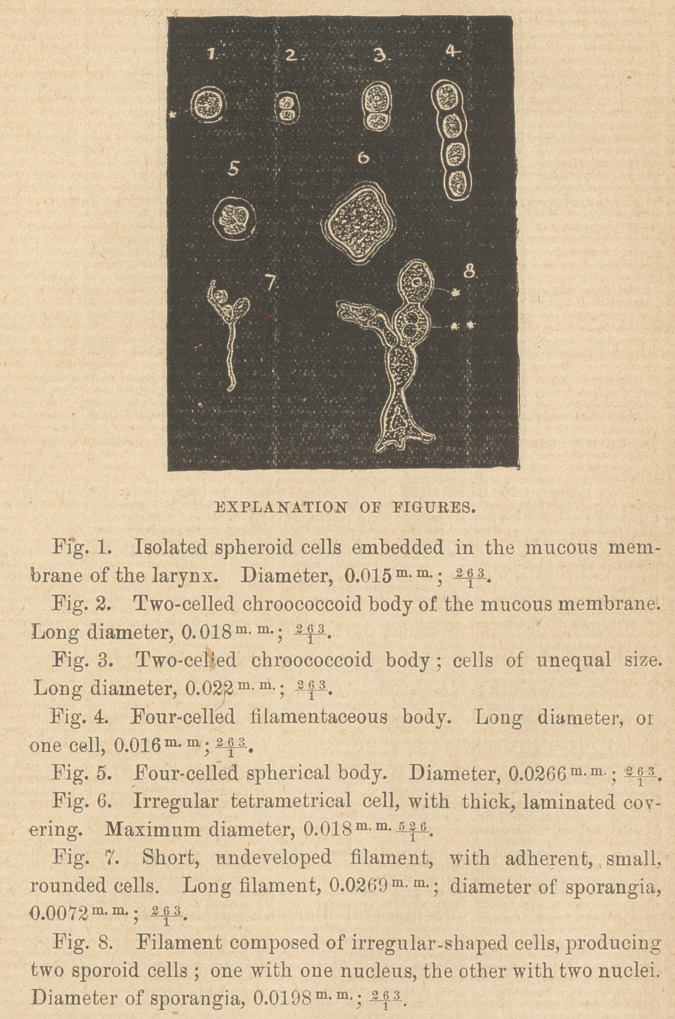


**Figure f2:**
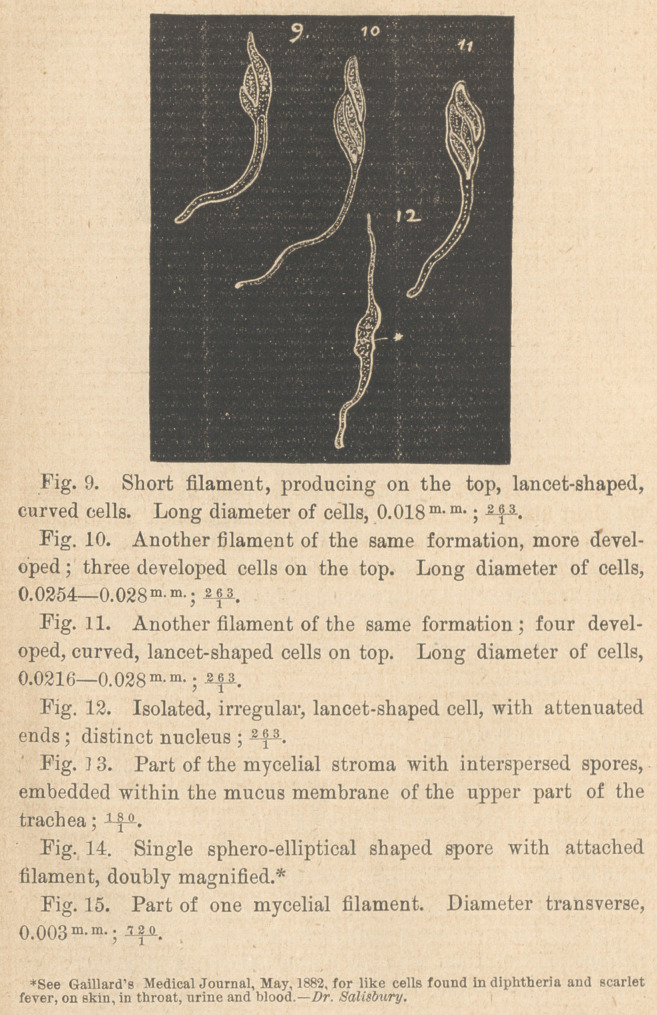


**Figure f3:**